# MiR-876-5p regulates gastric cancer cell proliferation, apoptosis and migration through targeting WNT5A and MITF

**DOI:** 10.1042/BSR20190066

**Published:** 2019-06-28

**Authors:** Zhenglei Xu, Zhichao Yu, Qinghong Tan, Cheng Wei, Qi Tang, Lisheng Wang, Yingcai Hong

**Affiliations:** 1 Department of Gastroenterology, The Second Clinical Medical College (Shenzhen People’s Hospital), Jinan University, Shenzhen 518000, Guangdong, China; 2 Department of Thoracic Surgery, The Second Clinical Medical College (Shenzhen People’s Hospital), Jinan University, Shenzhen 518000, Guangdong, China

**Keywords:** gastric cancer, miR-876-5p, MITF, WNT5A

## Abstract

MicroRNAs (miRNAs) are reported to play critical roles in various cancers. Recently, mounting miRNAs are found to exert oncogenic or tumor inhibitory role in gastric cancer (GC), however, their potential molecular mechanism in GC remains ill-defined. Currently, we aimed to elucidate the functional and mechanistic impacts of a novel miRNA on GC cellular process. The significant down-regulation of miR-876-5p in GC cells attracted our attention. In function, we performed gain-of-function assays and found that miR-876-5p overexpression repressed proliferative, anti-apoptotic and migratory abilities and epithelial–mesenchymal transition (EMT) of GC cells. By applying bioinformatics prediction and mechanism experiments, we verified that miR-876-5p could double-bind to the 3′ untranslated regions (3′UTRs) of Wnt family member 5A (WNT5A) and melanogenesis associated transcription factor (MITF), thus regulating their mRNA and protein levels. Both WNT5A and MITF were highly expressed in GC cells. Additionally, we conducted loss-of-function assays and confirmed the oncogenic roles of WNT5A and MITF in GC. Finally, rescue assay uncovered a fact that miR-876-5p suppressed GC cell viability and migration, but induced cell apoptosis via targeting WNT5A and MITF. Taken together, we might offer a valuable evidence for miR-876-5p role in GC development.

## Introduction

As one of the most frequent cancerous occurring in both male and female, gastric cancer (GC) is ranked third in cancer-related mortality all around the world [1,2]. According to the previous studies, a majority of GC patients were diagnosed in the advanced stage, thereby missing the optimal treatment time for radical gastrectomy [3,4]. In spite of great advances in surgical and comprehensive therapies, the clinical effect of GC patients remains to be improved [5,6]. It is well-known that genetic and epigenetic dysregulations are implicated in GC onset and progression [7,8]. Hence, the investigation of novel diagnostic and prognostic biomarkers in GC has been a research hotspot.

Recent advances in high-throughput genome sequencing technologies have provided a large amount of evidence that most of human transcriptome are non-coding RNAs (ncRNAs) without apparent protein-coding potential, including long ncRNAs, microRNAs (miRNAs), circular RNAs, pseudogenes, etc. [9]. Copious reports reveal that miRNAs are widely involved in various cancerous biological behaviors and pathological processes, including GC [10–12]. By completely or incompletely base-pairing with the 3′ untranslated region (3′UTR) of target messenger RNA (mRNA), miRNA triggers either mRNA degradation or translational repression [13–15]. Compelling evidence has indicated that miRNA is emerging as an indispensable modulator of cellular process, including cell proliferation, migration, apoptosis, differentiation, angiogenesis and immune response in various human diseases, especially human cancers [16–20]. Previously published studies have revealed that aberrantly expressed miRNAs are implicated in GC tumorigenesis, progression and drug resistance [21]. Ding et al. reported that miR-375 frequently down-regulated in GC suppressed cell proliferation through targeting JAK [22]. Shang et al. pointed out that miR-508-5p affects multidrug resistance of GC via regulating ABCB1 and ZNRD1, thereby forming an miR-508-5p/ZNRD1/ABCB1 regulatory loop [23]. Liu et al. unveiled a factor that miR-30a inhibits helicobacter pylori-related GC biological process by double-targeting COX-2 and BCL9 [24]. In addition to its antitumor role, miRNAs may also exert oncogenic effects on GC cellular processes, as exemplified by miR-27a which is commonly overexpressed in GC and promotes proliferation and metastasis of GC cells via inhibiting PHLPP2 and activating AKT/GSK3β pathway [25].

In the current study, we aimed to exploring the biological function and underlying mechanistic involvement of a novel miRNA in GC. Firstly, we selected miR-876-5p as a study focus after qRT-PCR examination. The following gain-of-function assays uncovered that miR-876-5p impaired proliferative, anti-apoptotic and migratory capacities of GC cells, acting as a tumor suppressor. Mechanistically, both Wnt family member 5A (WNT5A) and melanogenesis-associated transcription factor (MITF) were verified as the targets of miR-876-5p in GC. Importantly, WNT5A and MITF regulated positively GC cell viability and migration. Finally, rescue assays suggested that miR-876-5p regulated GC progression through targeting WNT5A and MITF.

## Materials and methods

### Cell culture

Three human GC cell lines (MGC803, MKN-45, MKN-28) and one normal human gastric epithelial cell line GES-1 were purchased from American Type Culture Collection (ATCC, Manassas, VA, U.S.A.). All cell lines were incubated in Dulbecco’s Modified Eagle’s Medium (DMEM; Invitrogen, Carlsbad, CA, U.S.A.). Medium was supplemented with 10% fetal bovine serum (FBS; Life Technologies, Carlsbad, U.S.A.) and 1% penicillin/streptomycin (Life Technologies). The culture condition in incubator was set as 37°C with 5% CO_2_. Culture medium was replaced every 3 days. Cells were passaged at approximately 80% confluence.

### Transfection

MGC803 and MKN-45 cells were incubated in six-well plate for 24 h before transfection. MiR-876-5p mimics (termed miR-876-5p) and control (NC) were synthesized and purchased from Sangon Biotech (Shanghai, China). For the overexpression of WNT5A and MITF, the pcDNA3.1 vector containing WNT5A and MITF (termed WNT5A and MITF) was synthesized and purchased from Sangon Biotech. Empty vector pcDNA3.1 was considered as the control. Small interfering RNAs (siRNAs) against WNT5A and MITF (si-WNT5A#1/2/3 and si-MITF#1/2/3) and control (si-NC) from Sangon Biotech were transfected into cells. The si-RNAs target sequences were indicated as follows: si-WNT5A#1 5′ ATGAAGAAGTCCATTGGAATATT 3′; si-WNT5A#2 5′ AAGAAACTGTGCCACTTGTATCA 3′; si-WNT5A#3 5′ CAGTGATTCTGGTTTTTGGTTTT 3′; si-MITF#1 5′ TGGGAAGTTACTGTTACTTGATA 3′; si-MITF#2 5′ AGGCTTTCTAGAAAGAATAAACT 3′; si-MITF#3 5′ TTGAACGAAGAAGAAGATTTAAC 3′; sh-NC 5′ GTTCTCCGAACGTGTCACGT 3′. The cell transfection was carried out using Lipofectamine2000 (Invitrogen, Carlsbad, CA, U.S.A.) according to the standard method. 48-h later, cells were harvested. Cell transfection was carried out at least three times.

### Quantitative real-time PCR

Total RNA was isolated from cells using a mirVana™ miRNA Isolation Kit (Thermo Fisher Scientific, Waltham, MA, U.S.A.) in accordance with the user guide. The complementary DNA (cDNA) synthesis was obtained using a High-Capacity RNA‐to‐cDNA™ Kit (Thermo Fisher Scientific, Waltham, MA, U.S.A.). The quantitative real-time PCR (qRT-PCR) reaction was carried out using the miScript SYBR Green PCR Kit (TransGen Biotech, Beijing, China). The qRT-PCR thermocycling was conducted with a denaturation step of 2 min at 95°C, 40 cycles at 95°C for 15 s and 60°C for 30 s. The expression levels of genes were normalized to U6 or GAPDH, using 2^−ΔΔCt^ method. The primers were shown as follows: miR-876-5p, forward 5′-TGAAGTGCTGTGGATTTCTTTGTG-3′ and reverse 5′-TGAATTACTTTGTAAACCACCACCA-3′; WNT5A, forward 5′-GCTCTTCAGAAGGAACCATTGC-3′ and reverse 5′-TCTGCTTTCACCCAGTAGGC-3′; MITF, forward 5′-TCTGTTCTCACTTTCCAGCAGT-3′ and reverse 5′-CTGTCACCACTCACCTGCTCTT-3′; GAPDH, forward 5′-CGGAGTCAACGGATTTGGTCGTATTGG-3′ and reverse 5′-GCTCCTGGAAGATGGTGATGGGATTTCC-3′; U6, forward 5′-GACGAATACCGGCGTGAGAA-3′ and reverse 5′-AAATTCTGTTTGCGGTGCGT-3′. qRT-PCR was carried out at least three times.

### Cell Counting Kit-8 assay

Cell Counting Kit-8 (CCK-8) assay was performed to assess cell proliferative ability. After transfection for 48 h, MGC803 and MKN-45 cells (5 × 10^3^) were incubated in 96-well plates at 37°C with 5% CO_2_ and respectively incubated for 24, 48, 72 and 96 h. Thereafter, each well was added with 10 μl CCK-8 (Dojindo, Japan), followed by incubation for 4 h. FLx800 Fluorescence Microplate Reader (Biotek, Winooski, VT, U.S.A.) was utilized to detect the absorbance values at 450 nm. CCK-8 assay was carried out at least three times.

### 5‐Ethynyl‐2′‐deoxyuridine‐labeling assay

The 5-ethynyl‐2′-deoxyuridine (EdU)-labeling assay was performed to detect GC cell proliferation ability. MGC803 and MKN-45 cells were incubated in 96-well (4 × 10^4^ cells per well) plates. Thereafter, cells were treated with 50 μM EdU and incubated for 2 h at 37°C. After washing with phosphate buffered saline (PBS) for two times, cells were fixed with 4% paraformaldehyde for 30 min at room temperature. Thereafter, cells were washed with PBS after treating with 50 μl glycine (2 mg/ml), decolorizing for 5 min. Cells were permeabilized by 0.5% Triton X-100 for 5 min. 100 μl 1× Apollo reaction cocktail was added and incubated for 30 min. Subsequently, 4,6-diamidino-2-phenylindole (DAPI) was utilized to stain the cell nuclei for 15 min. A fluorescence microscope (Olympus) was used to obtain images. EdU assay was performed more than two times.

### Caspase 3 activity assay

5 × 10^6^ MGC803 and MKN-45 cells were plated into 96-well plates. The transfected cells were re-suspended in 50 μl of chilled Cell Lysis Buffer. After separating cell supernatant, the protein concentration was assessed. Thereafter, specimens were treated with 50 μl of 2 × Reaction Buffer containing 5 μl of 4 mM DEVD-p-NA substrate (200 μM final concentration) and incubated at 37°C for 4 h. Finally, the absorbance of reaction mixture was measured by microplate reader (Tecan Group Ltd., Männedorf, Switzerland) at the wavelength of 405 nm. All experimental procedures were thrice repeated independently.

### Flow cytometer analysis

Treated GC cells were trypsinized, washed twice with cold PBS and resuspended in Annexin V Binding Buffer, followed by double staining with FITC Annexin V and propidiumiodide (PI) using the Annexin V FITC apoptosis detection kit (BD Bioscience, CA, U.S.A.) as instructed by the manufacturer. The apoptotic cells were examined on a flow cytometer (BD Bioscience).

### Transwell assay

Transwell assay was conducted and perform to investigate GC cell migratory ability. In brief, 200 μl MGC803 and MKN-45 were seed into the upper Transwell chamber (8-μm pores, Corning, NY, U.S.A.). The lower chamber contained 500 μl DMEM mixed with 10% FBS (Gibco, U.S.A.). After incubating for 24 h, non-migrating cells on the upper surface of the chamber were removed using cotton swab. Migratory cells on the lower surface of the chamber were blocked with paraformaldehyde for 30 min and stained with 0.1% crystal violet for 20 min. Finally, migratory cells were determined using a microscopy (×200 magnification; Nikon, Japan) and counted in five random fields. This experiment was performed at least three times.

### Immunofluorescence staining

Treated GC cells were placed into 24-well plates and fixed with 4% paraformaldehyde and permeabilized with 100 μM digitonin, and blocked by 1% BSA for 30 min, followed by overnight incubation at 4°C with primary antibodies against E-cadherin (1:100, ab15148, Abcam, Cambridge, MA, U.S.A.), N-cadherin (1:100, ab18203, Abcam), Vimentin (1:100, ab137321, Abcam), α-SMA (1:100, ab32575, Abcam) and Snail (1:100, ab180714, Abcam). Then cells were washed in PBS prior to incubation at room temperature for 1 h with Alexa Fluor® 488 Goat Anti-Rabbit IgG H&L (1:200, ab150077, Abcam) and subsequently stained with DAPI (Cell Signaling Technology, Danvers, MA, U.S.A.). Cells were visualized and photographed under LSM 800 with Airyscan (Zeiss, Jena, Germany).

### Luciferase reporter assay

Luciferase reporter assay was performed in MGC803 and MKN-45 cells. The wild type of WNT5A and MITF (WNT5A-WT and MITF-WT) and mutant type of WNT5A and MITF (WNT5A-MUT and MITF-MUT) were synthesized and purchased from Sangon Biotech (Shanghai, China). Thereafter, WNT5A-WT/MUT and MITF-WT/MUT were respectively co-transfected into MGC803 and MKN-45 cells along with miR-876-5p or miR-NC using Lipofectamine2000 (Invitrogen) in accordance with the user guide. After 48-h transfection, cells were reaped. The luciferase activity of RNAs was detected by the dual luciferase reporter assay system (Promega, Madison, Wisconsin, U.S.A.) and normalized to that of the Renilla luciferase. Luciferase reporter assay was carried out at least three times.

### Pull-down assay

Biotinylated RNA probes (Bio-miR-NC, Bio-miR-876-5p-WT, Bio-miR-876-5p-Mut) were synthesized by Thermo Fisher Scientific (Waltham, MA, U.S.A.). MGC803 and MKN-45 cells were lysed with the radio immunoprecipitation assay (RIPA) lysis buffer (Beyotime, Shanghai, China) mixed with RNase inhibitor (Invitrogen). Thereafter, cell lysates were incubated with aforementioned RNA probes for 1 h at 4°C, treated with 50 μl of streptavidin beads for 30 min at 4°C. Subsequently, Biotin Elution Buffer was utilized to elute the RNA–protein complex. Finally, the RNA–protein complex was detected by qRT-PCR assay. Pull-down assay was thrice performed.

### Western blot analysis

Total protein was isolated from cells using RIPA lysis buffer (Beyotime, Shanghai, China) mixed with protease inhibitors. The concentration of isolated protein was measured using the BCA Kit (Thermo Scientific Pierce). 50 μg of isolated protein was separated by 10% sodium dodecyl sulfate-polyacrylamide gel electrophoresis (SDS-PAGE) and transfected to a polyvinylidene fluoride (PVDF) membrane (Millipore, Billerica, MA, U.S.A.). Afterwards, membranes were sealed with Tris-buffered saline (TBS) mixed with 5% skimmed milk. The membrane was incubated with primary antibodies at 4°C overnight. The primary antibodies were listed as follows: Bax (1:1000, #ab32503), Bcl-2 (1:1000, #ab32124), E-cadherin (1:1000, #ab76055), N-cadherin (1:1000, #ab18203), WNT5A (1:1000, #ab32199), MITF (1:200, #ab140606) and GAPDH (1:1000, #ab8245). All primary antibodies were purchased from Abcam (Cambridge, MA, U.S.A.). After washing, membranes were incubated with horseradish peroxidase-conjugated secondary antibody (1:2000, Santa Cruz Co, Dallas, TX, U.S.A.) for 1 h at room temperature. Finally, protein signals were detected using enhanced chemiluminescence (ECL; Pierce, Rockford, IL, U.S.A.). GAPDH was considered as internal control. Western blotting was carried out at least three times.

### Bioinformatics analysis

The prediction tools (RNA22, PITA, miRmap, miRanda and microT) from StarBase (http://starbase.sysu.edu.cn/index.php) utilized to screen out the targets of miR-876-5p. The putative binding sites between miR-876-5p and WNT5A and MITF were acquired from Starbase.

### Xenografts experiments

Female 6-week-old athymic BABL/c nude mice commercially obtained from the Animal Center of Guangdong Province (Guangzhou, China) were randomly distributed to four groups (three per group) with different transfections. The logarithmic phase MGC803 cells (5 × 10^4^) with different transfections were injected into tail vein of the nude mice with sterile 28 gauge needles for detecting the tumorigenesis. The tumors were carefully removed from the bodies of the killed mice after 28 days. Tumor weight and volume were measured, respectively.

### Statistical analysis

All data were obtained from at least three replications. Data were showed as mean ± standard deviation (SD). The statistical analyses were performed using SPSS version 17.0 software (Abbott Laboratories, Chicago, IL, U.S.A.). A two-sided Student’s *t*-test and one-way analysis of variance were utilized to ascertain differences between two groups or more than two groups. *P* < 0.05 was considered statistical significance.

## Results

### MiR-876-5p up-regulation suppressed GC cell viability and migration, but induced cell apoptosis

**Figure 1:**
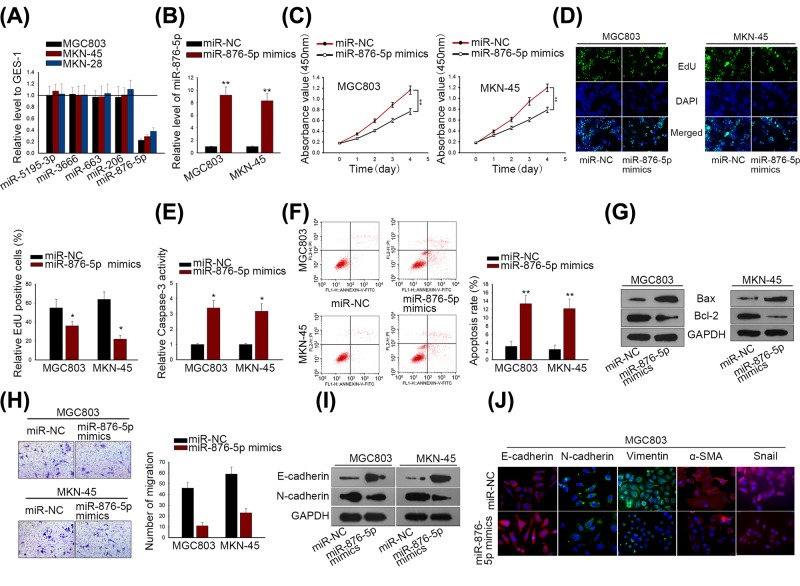
MiR-876-5p up-regulation suppressed GC cell viability and migration, but induced cell apoptosis (**A**) The five acknowledged miRNAs (miR-5195-3p, miR-3666, miR-23c, miR-4429 and miR-876-5p) were chosen to qRT-PCR. (**B**) MiR-876-5p expression was overexpressed in MGC803 and MKN-45 cells. (**C,D**) GC cell proliferation was measured by CCK-8 and EdU assays. (**E**) Caspase-3 activity was detected to evaluate the GC cell apoptosis in response to miR-876-5p overexpression. (**F**) Apoptotic cell ratio in MGC803 and MKN-45 was detected by flow cytometric analysis. (**G**) Western blot assay was used to assess the level of apoptosis-related protein (Bcl-2 and Bax). (**H**) Transwell migration assay was conducted in MGC803 and MKN-45 cells. (**I**) The alteration of EMT-associated protein (E-cadherin and N-cadherin) level was analyzed by Western blotting. (**J**) Immunofluorescence staining examined the different expression levels of EMT biomarkers in MGC803 transfected with miR-NC or miR-876-5p mimics. **P < 0.01 and *P < 0.05 vs control group.

Firstly, five acknowledged miRNAs (miR-5195-3p, miR-3666, miR-23c, miR-4429 and miR-876-5p), which have been reported as tumor suppressors in various cancers [26–30], were selected to perform qRT-PCR. Results indicated that only miR-876-5p was significantly down-regulated in GC cells compared with the normal human gastric epithelial cell line GES-1 (Figure 1A). Hence, we focused on miR-876-5p in subsequent analysis. Among GC cells, miR-876-5p was lowly expressed in MKN-45 and MGC803. With that, MGC803 and MKN-45 cells were transfected with miR-876-5p mimics or miR-NC (Figure 1B). CCK-8 and EdU assays indicated that the miR-876-5p up-regulation significantly reduced MGC803 and MKN-45 cell viability compared with control groups (Figure 1C,D). The enhanced miR-876-5p expression obviously increased the activity of caspase-3 in MGC803 and MKN-45 cells (Figure 1E) and augmented apoptotic cell ratio (Figure 1F), showing that miR-876-5p induced more apoptotic cells in GC. In addition, we measured the levels of apoptosis-associated proteins (Bax and Bcl-2) in MGC803 and MKN-45 cells. Results of Western blot suggested that miR-876-5p mimics significantly increased the protein level of Bax, but decreased that of Bcl-2 (Figure 1G). Transwell assay uncovered that miR-876-5p overexpression significantly inhibited the migratory ability of MGC803 and MKN-45 cells (Figure 1H). Besides, we detected the levels of epithelial–mesenchymal transition (EMT)-related proteins (E-cadherin and N-cadherin) in GC cells. It was observed that miR-876-5p mimics hindered EMT process via strengthening E-cadherin expression and lessening the expression of N-cadherin, Vimentin, α-SMA, Snail (Figure 1I,J). Through these functional assays, we came to the conclusion that miR-876-5p inhibited GC progression, acting as a tumor suppressor.

### MiR-876-5p targeted WNT5A and MITF in GC cells

**Figure 2:**
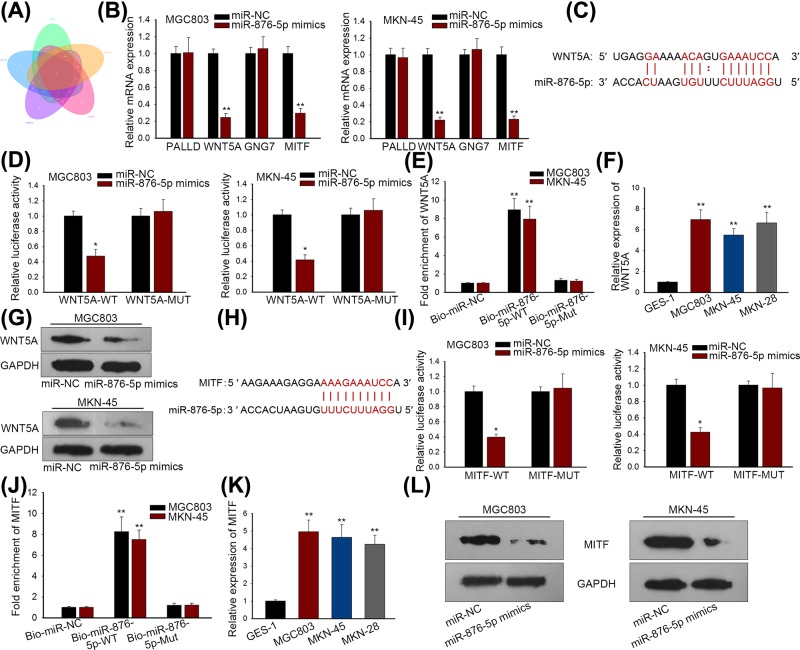
MiR-876-5p targeted WNT5A and MITF in GC cells (**A**) Venn diagram showed the predicted four targets of miR-876-5p. (**B**) qRT-PCR revealed that WNT5A and MITF were significantly down-regulated in response to miR-876-5p mimics. (**C**) The putative binding sites of miR-876-5p in 3′UTR of WNT5A. (**D,E**) Luciferase reporter and RNA pull-down assays were applied to verify the binding of miR-876-5p in 3′UTR of WNT5A. (**F**) WNT5A was up-regulated in GC cell lines. (**G**) WNT5A protein level was reduced by miR-876-5p overexpression in GC cells. (**H**) The predicted binding sequences of miR-876-5p in 3′UTR of MITF. (**I,J**) Luciferase reporter and RNA pull-down assays were applied to verify the binding of miR-876-5p in 3′UTR of MITF. (**K**) MITF was highly expressed in GC cell lines. (**L**) MiR-876-5p repressed the protein level of MITF. **P < 0.01 and *P < 0.05 vs control group.

Through the intersection of five bioinformatics prediction tools (PITA, miRanda, mircoT, miRmap and RNA22) from Starbase, we screened four putative targets (PALLD, WNT5A, GNG7 and MITF) for miR-876-5p. Venn diagram was shown in Figure 2A. Results of qRT-PCR disclosed that expression levels of both WNT5A and MITF were significantly weakened by miR-876-5p overexpression in MGC803 and MKN-45 cells, while no significant change in levels of PALLD and GNG7 was observed on introduction of miR-876-5p mimics (Figure 2B). Thus we selected WNT5A and MITF as main targets for subsequent assays. The binding sites between miR-876-5p and WNT5A were displayed in Figure 2C. Luciferase reporter assay revealed that miR-876-5p mimics lessened the luciferase activity of wild type WNT5A (WNT5A-WT), but not that of mutant form WNT5A (WNT5A-MUT) in MGC803 and MKN-45 cells (Figure 2D). Additionally, pull-down assay showed that the biotinylated miR-876-5p (Bio-miR-876-5p-WT) probe enhanced the level of WNT5A than Bio-miR-NC and Bio-miR-876-5p-Mut probes (Figure 2E). Importantly, WNT5A was noticeably up-regulated in GC cells (Figure 2F). Result from western blotting indicated that miR-876-5p overexpression repressed WNT5A protein level in GC cells (Figure 2G). Moreover, the binding sites between miR-876-5p and MITF were shown in Figure 2H. Similarly, luciferase reporter and RNA pull down assays confirmed the binding of miR-876-5p to the 3′UTR of MITF in MGC803 and MKN-45 cells (Figure 2I,J). The up-regulated expression of MITF was found in GC cell lines (Figure 2K). More importantly, miR-876-5p modulated negatively MITF protein level in GC cells (Figure 2L). Thus, we identified WNT5A and MITF as targets of miR-876-5p in GC.

### Both WNT5A and MITF functioned as oncogenes in regulating GC biological process

**Figure 3:**
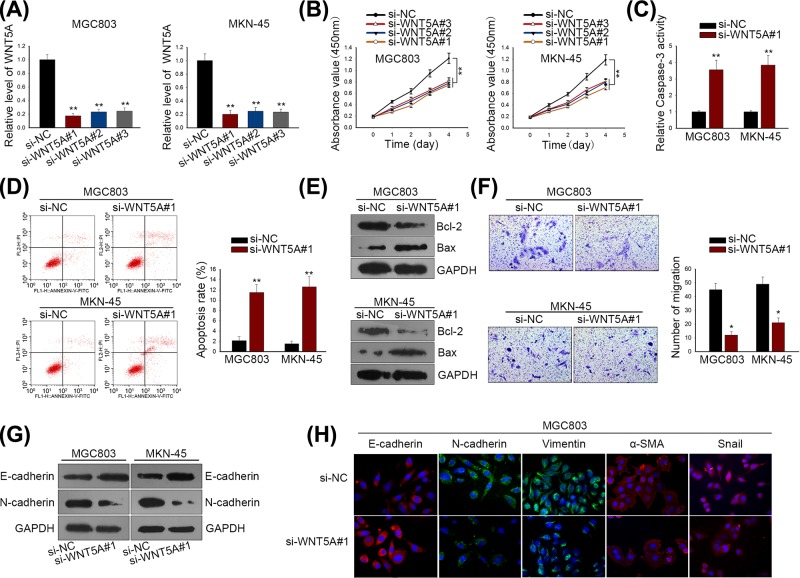
WNT5A functioned as oncogenes in regulating GC biological process (**A**) WNT5A expression was silenced by si-WNT5A#1/2/3 in both MGC803 and MKN-45 cells. (**B**) CCK-8 assay was used to measure GC cell proliferation after silencing WNT5A expression. (**C**) Caspase-3 activity was detected for GC cell apoptosis. (**D**) Apoptotic cells in MGC803 and MKN-45 cells were determined by flow cytometry. (**E**) Western blot analysis for apoptosis-related proteins (Bcl-2 and Bax). (**F**) GC cell migratory ability was evaluated by Transwell migration assay. (**G**) Western blotting for EMT-associated proteins (E-cadherin and N-cadherin). (**H**) Immunofluorescence staining examined the different expression levels of EMT biomarkers MGC803 cells transfected with si-NC or si-WNT5A#1. **P < 0.01 and *P < 0.05 vs control group.

**Figure 4:**
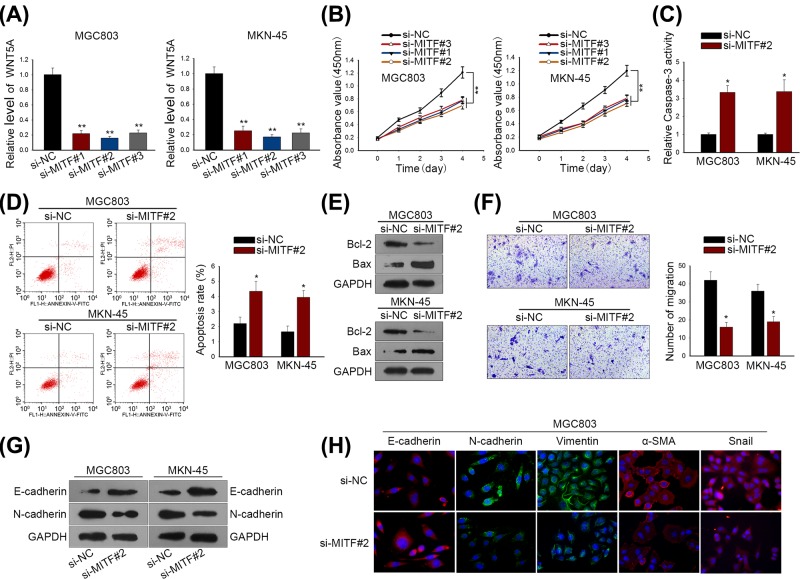
MITF functioned as oncogenes in regulating GC biological process (**A**) MITF expression was silenced by si-MITF#1/2/3 in MGC803 and MKN-45 cells. (**B**) CCK-8 assay was used to measure GC cell proliferation in response to MITF depletion. (**C**) Caspase-3 activity was detected for GC cell apoptosis. (**D**) Apoptotic cells in MGC803 and MKN-45 were determined by flow cytometry. (**E**) Western blot analysis for apoptosis-related proteins (Bcl-2 and Bax). (**F**) GC cell migratory ability was evaluated by Transwell migration assay. (**G**) Western blotting for EMT-associated proteins (E-cadherin and N-cadherin). (**H**) Immunofluorescence staining examined the different expression levels of EMT biomarkers in MGC803 cells transfected with si-NC or si-MITF#2. **P < 0.01 and *P < 0.05 vs control group.

Subsequently, we intended to further evaluate the bio-function of WNT5A and MITF in GC cells. Using specific siRNAs (si-WNT5A#1/2/3 and si-MITF#1/2/3), we silenced expressions of WNT5A and MITF in MGC803 and MKN-45 cells, wherein si-WNT5A#1 and si-MITF#2 exhibited the best knockdown efficacy, respectively (Figures 3A and 4A). CCK-8 assay disclosed a fact that GC cell viability was remarkably weakened by si-WNT5A#1/2/3 and si-MITF#1/2/3, especially by si-WNT5A#1 and si-MITF#1 (Figures 3B and 4B). Next, we estimated the effect of si-WNT5A#1 and si-MITF#1 on GC cell apoptosis and migration. As shown in Figures 3C,D and 4C,D, the knockdown of WNT5A and MITF induced more apoptotic cells in MGC803 and MKN-45 cell lines. Besides, result of Western blotting indicated that the silenced WNT5A and MITF expressions noticeably enhanced the level of Bcl-2 protein, but lessened the level of Bax protein (Figures 3E and 4E). Transwell assay indicated that migratory capacity of MGC803 and MKN-45 cells was suppressed by the depleted WNT5A and MITF expressions (Figures 3F and 4F). Moreover, we detected the alteration of EMT-associated protein levels by Western blot and immunofluorescence. It was found that depletions of WNT5A and MITF reversed EMT into MET through strengthening E-cadherin expression and reducing levels of mesenchymal markers (Figures 3G,H and 4G,H). Hence, we concluded that both WNT5A and MITF exerted pro-tumorigenesis role in GC cellular process.

### MiR-876-5p inhibited GC cellular process via targeting WNT5A and MITF *in vitro*

**Figure 5:**
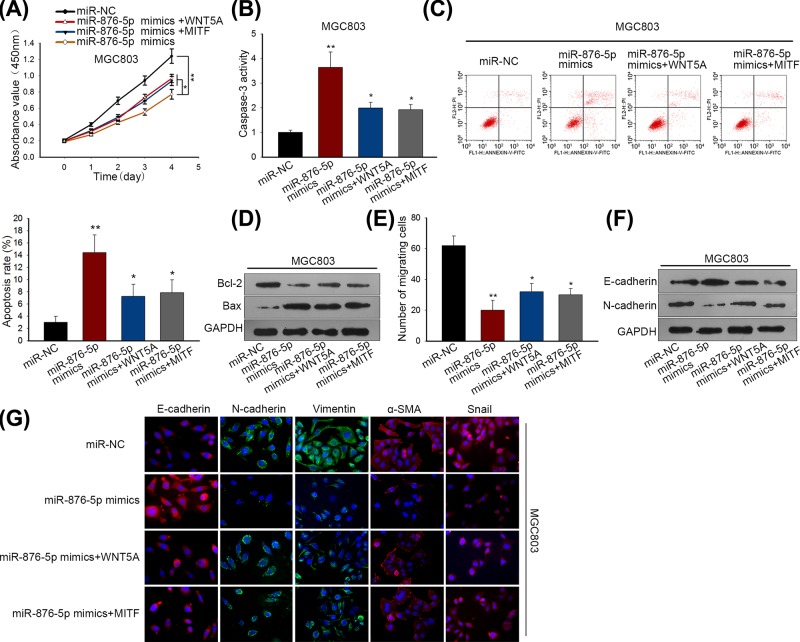
MiR-876-5p inhibited GC cellular process via targeting WNT5A and MITF (**A**) GC cell proliferative capacity was assessed by CCK-8 assay in MGC803 cell after transfection with miR-NC, miR-876-5p mimics, miR-876-5p mimics + WNT5A or miR-876-5p mimics + MITF. (**B,C**) Caspase-3 activity and flow cytometry assays indicated that the positive impact of miR-876-5p mimics on GC cell apoptosis was reversed by pcDNA-WNT5A and pcDNA-MITF. (**D**) The level alteration of Bcl-2 and Bax after treatment with various transfection plasmids. (**E**) The effect of the enhanced WNT5A and MITF expressions on GC cell migration in miR-876-5p-up-regulated MGC803 cell. (**F**) EMT process was promoted by miR-876-5p overexpression, but this acceleration was weakened by overexpression of WNT5A and MITF. (**G**) Immunofluorescence staining examined the different expression levels of EMT biomarkers in MGC803 cells transfected with miR-NC, miR-876-5p mimics, miR-876-5p mimics + WNT5A or miR-876-5p mimics + MITF. **P < 0.01 and *P < 0.05 vs control group.

At length, we designed and performed a series of functional rescue assays to confirm whether miR-876-5p could modulate GC biological behaviors through WNT5A and MITF. Significantly, the enhanced WNT5A and MITF expressions promoted GC cell proliferation in miR-876-5p-overexpressed MGC803 cell line (Figure 5A). Caspase-3 activity and cell apoptosis was facilitated by miR-876-5p mimics, but mitigated by overexpression of WNT5A and MITF (Figure 5B,C). In addition, the reduced level of Bcl-2 by miR-876-5p up-regulation was impaired through transfection MGC803 cell with pcDNA-WNT5A and pcDNA-MITF, whereas we observed an opposite impact on Bax protein level (Figure 5D). MiR-876-5p mimics inhibited GC cell migration, but the enhanced WNT5A and MITF expressions abrogated this inhibitory effect (Figure 5E). Importantly, overexpression of WNT5A and MITF accelerated EMT process in miR-876-5p-up-regulated MGC803 cell (Figure 5F,G). Through all findings, we found that miR-876-5p regulated negatively GC cellular process via targeting WNT5A and MITF.

### MiR-876-5p restrained GC cell proliferation via targeting WNT5A and MITF *in vivo*

**Figure 6:**
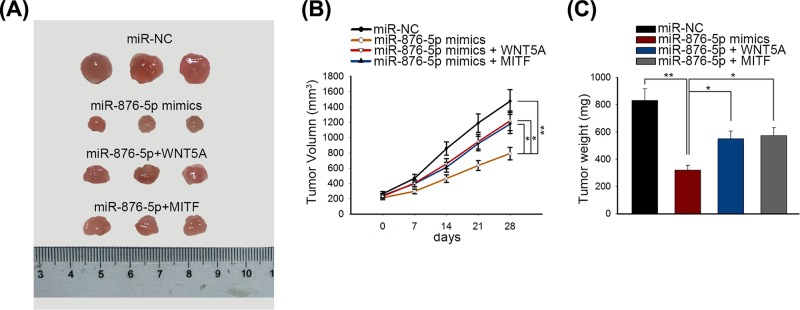
MiR-876-5p inhibited tumor growth of GC *in vivo* (**A**) The sizes of tumors derived from mice of different groups (mimics control group, miR-876-5p mimics group, miR-876-5p + WNT5A group, miR-876-5p + MITF group) were measured. (**B,C**) The tumor volume and tumor weight were separately measured after 28 days. Tumor volume was measured every week. **P < 0.01 and *P < 0.05 vs control group.

To investigate whether miR-876-5p affected GC development *in vivo*, treated MGC803 cells were injected into the tail vein of nude mice. Tumors derived from different groups were observed and photographed. Tumors from the mice injected with miR-876-5p-transfected MGC803 cells were smaller than that derived from mimics control-transfected mice; in miR-876-5p and si-WNT5A or si-MITF group, overexpression of WNT5A or MITF mitigated the inhibitory effects of miR-876-5p on tumor size (Figure 6A). Consistently, compared with the mimics control group, the tumor volume and tumor weight in the miR-876-5p mimics group were significantly reduced, which can be rescued by ectopic expression of WNT5A or MITF (Figure 6B,C).

## Discussion

Although the early diagnosis and therapy of GC were rapidly developed over the past decades, most of the patients with GC were faced with metastasis recurrence [31–33]. It is important for GC patients to search out effective new biomarkers. Recently, miRNA is emerging as a vital regulator, participating in GC development. miRNA-876-5p has been frequently reported as a tumor suppressor in different types of cancer. In hepatocellular carcinoma (HCC), miR-876-5p was revealed to enhance sensitivity to sorafenib and restrain HCC cell proliferation, migration and EMT progression by repression on its target genes [34–36]. In addition, miR-876-5p was reported to assume an antitumor role in lung cancer [37], head and neck squamous cell carcinoma [38], and osteosarcoma [39]. The other mature isoform of miR-876, miR-876-3p has been reported to suppresse the durg-resistance of GC cells [40]. However, effect of miR-876-5p on GC progression is still unknown. Hence, we focused on miR-876-5p in the present study.

In our study, up-regulation of miR-876-5p in three GC cell lines was determined by qRT-PCR. In addition, gain-of-function assays uncovered a fact that miR-876-5p overexpression significantly inhibited cell proliferation, migration and EMT progress, but induced more apoptotic cells in GC. Noticeably, miR-876-5p acted as a tumor repressor in GC via regulating cell proliferation, migration and apoptosis.

To probe into the downstream target genes of miR-876-5p, we applied bioinformatics prediction tools. Among all candidate targets, qRT-PCR disclosed that only WNT5A and MITF were obviously down-regulated in response to miR-876-5p overexpression in GC cells. Of significance, we found the up-regulated WNT5A and MITF expressions. Moreover, it was observed that WNT5A and MITF functioned as targeted mRNAs of miR-876-5p, thus exerting positively functional role in affecting GC cellular process (proliferation, migration and apoptosis). Previous studies have unveiled the oncogenic role of WNT5A and MITF in GC. Kurayoshi et al. unmasked that WNT5A exacerbates the aggressiveness of GC cells [41]. Xue et al. reported that WNT5A is overexpressed in GC and integral to ZEB1-induced progression of GC [42]. Yingduan et al. uncovered that MITF as an oncogene in GC cells was repressed via chromatin modification by ZNF382 [43]. According to the existing studies, WNT5A was identified as a non-canonical ligand in Wnt family, with high expression in several human malignancies including GC [44–46]. It is noteworthy that WNT5A could activate the β-catenin-mediated Wnt signaling pathway, thereby driving cancerous progression [47]. Furthermore, as described in previous reports, MITF was similarly linked to Wnt/β-catenin signaling pathway [48], which is considered as one of representative target genes of β-catenin [49]. Therefore, whether miR-876-5p can modulate GC initiation and development via modulating Wnt/β-catenin signaling pathway remains to be further explored in the future.

Summing up the above, we reported the inhibitory role of miR-876-5p in affecting GC biological behaviors, including cell proliferation, migration, EMT process and apoptosis. In mechanism, miR-876-5p targeted WNT5A and MITF, thus exerting functional regulation in GC cells. To our knowledge, this original study might be the first report for the role of miR-876-5p in GC, providing a potential biomarker for GC therapy.

## Ethic Statement

Animal care and euthanasia were subject to the Guide for the Care and Use of Laboratory Animals and approved by the review board of The Second Clinical Medical College (Shenzhen People’s Hospital).

## Abbreviations

CCK-8: Cell Counting Kit-8
DAPI: 4,6-diamidino-2-phenylindole
DMEM: Dulbecco’s Modified Eagle’s Medium
EdU: 5‐ethynyl‐2′‐deoxyuridine
EMT: epithelial–mesenchymal transition
FBS: fetal bovine serum
GC: gastric cancer
HCC: hepatocellular carcinoma
miRNA: microRNA
MITF: melanogenesis associated transcription factor
mRNA: messenger RNA
ncRNA: non-coding RNA
PBS: phosphate buffered saline
RIPA: radio immunoprecipitation assay
siRNA: small interfering RNA
WNT5A: Wnt family member 5A
3′UTR: 3′ untranslated region

## References

[1] Gong P., Qiao F., Wu H., Cui H., Li Y., Zheng Y. (2018) LncRNA UCA1 promotes tumor metastasis by inducing miR-203/ZEB2 axis in gastric cancer. Cell Death Dis. 9, 1158 30464170 10.1038/s41419-018-1170-0 PMC6249325

[2] Li Y., Zeng C., Hu J., Pan Y., Shan Y., Liu B. (2018) Long non-coding RNA-SNHG7 acts as a target of miR-34a to increase GALNT7 level and regulate PI3K/Akt/mTOR pathway in colorectal cancer progression. J. Hematol. Oncol. 11, 89 29970122 10.1186/s13045-018-0632-2 PMC6029165

[3] Zou Z., Ma T., He X., Zhou J., Ma H., Xie M. (2018) Long intergenic non-coding RNA 00324 promotes gastric cancer cell proliferation via binding with HuR and stabilizing FAM83B expression. Cell Death Dis. 9, 717 29915327 10.1038/s41419-018-0758-8 PMC6006375

[4] Hong D., Zhang X., Li R., Yu J., Lou Y., He Q. (2018) Deletion of TMEM268 inhibits growth of gastric cancer cells by downregulating the ITGB4 signaling pathway. Cell Death Differ. 10.1038/s41418-018-0223-3 PMC6748091 30361615

[5] Cao J., Lv W., Wang L., Xu J., Yuan P., Huang S. (2018) Ricolinostat (ACY-1215) suppresses proliferation and promotes apoptosis in esophageal squamous cell carcinoma via miR-30d/PI3K/AKT/mTOR and ERK pathways. Cell Death Dis. 9, 817 30050135 10.1038/s41419-018-0788-2 PMC6062526

[6] Gu Z., Li Y., Yang X., Yu M., Chen Z., Zhao C. (2018) Overexpression of CLC-3 is regulated by XRCC5 and is a poor prognostic biomarker for gastric cancer. J. Hematol. Oncol. 11, 115 30217218 10.1186/s13045-018-0660-y PMC6137920

[7] Cheng A.S., Li M.S., Kang W., Cheng V.Y., Chou J.L., Lau S.S. (2013) Helicobacter pylori causes epigenetic dysregulation of FOXD3 to promote gastric carcinogenesis. Gastroenterology 144, 122.e9–133.e9 10.1053/j.gastro.2012.10.002 23058321

[8] Palanisamy N., Ateeq B., Kalyana-Sundaram S., Pflueger D., Ramnarayanan K., Shankar S. (2010) Rearrangements of the RAF kinase pathway in prostate cancer, gastric cancer and melanoma. Nat. Med. 16, 793–798 10.1038/nm.2166 20526349 PMC2903732

[9] Eidem T.M., Kugel J.F. and Goodrich J.A. (2016) Noncoding RNAs: regulators of the mammalian transcription machinery. J. Mol. Biol. 428, 2652–2659 10.1016/j.jmb.2016.02.019 26920110 PMC4894004

[10] Song J.H. and Meltzer S.J. (2012) MicroRNAs in pathogenesis, diagnosis, and treatment of gastroesophageal cancers. Gastroenterology 143, 35.e2–47.e2 10.1053/j.gastro.2012.05.003 22580099

[11] Zhang J.X., Xu Y., Gao Y., Chen C., Zheng Z.S., Yun M. (2017) Decreased expression of miR-939 contributes to chemoresistance and metastasis of gastric cancer via dysregulation of SLC34A2 and Raf/MEK/ERK pathway. Mol. Cancer 16, 18 10.1186/s12943-017-0586-y 28114937 PMC5259972

[12] Kong P., Zhu X., Geng Q., Xia L., Sun X., Chen Y. (2017) The microRNA-423-3p-Bim axis promotes cancer progression and activates oncogenic autophagy in gastric cancer. Mol. Therapy: J. Am. Soc. Gene Therapy 25, 1027–1037 10.1016/j.ymthe.2017.01.013 28254439 PMC5383553

[13] Meng L., Liu F., Ju Y., Ding P., Liu S., Chang S. (2018) Tumor suppressive miR-6775-3p inhibits ESCC progression through forming a positive feedback loop with p53 via MAGE-A family proteins. Cell Death Dis. 9, 1057 30333480 10.1038/s41419-018-1119-3 PMC6193014

[14] Salem M., O’Brien J.A., Bernaudo S., Shawer H., Ye G., Brkic J. (2018) miR-590-3p promotes ovarian cancer growth and metastasis via a novel FOXA2-Versican pathway. Cancer Res. 78, 4175–4190 10.1158/0008-5472.CAN-17-3014 29748371

[15] Pu M., Chen J., Tao Z., Miao L., Qi X., Wang Y. (2018) Regulatory network of miRNA on its target: coordination between transcriptional and post-transcriptional regulation of gene expression. Cell. Mol. Life Sci. 10.1007/s00018-018-2940-7 PMC11105547 30374521

[16] Yi J.T., Chen T.T., Huo J. and Chu X. (2017) Nanoscale zeolitic imidazolate framework-8 for ratiometric fluorescence imaging of microRNA in living cells. Anal. Chem. 89, 12351–12359 10.1021/acs.analchem.7b03369 29083869

[17] Long M., Zhan M., Xu S., Yang R., Chen W., Zhang S. (2017) miR-92b-3p acts as a tumor suppressor by targeting Gabra3 in pancreatic cancer. Mol. Cancer 16, 167 10.1186/s12943-017-0723-7 29078789 PMC5659029

[18] Yang C., Ma X., Guan G., Liu H., Yang Y., Niu Q. (2018) MicroRNA-766 promotes cancer progression by targeting NR3C2 in hepatocellular carcinoma. FASEB J.: Off. Publication Feder. Am. Societies Exp. Biol fj201801151R 10.1096/fj.201801151R 30130435

[19] Yu X., Zhang Y., Cavazos D., Ma X., Zhao Z., Du L. (2018) miR-195 targets cyclin D3 and survivin to modulate the tumorigenesis of non-small cell lung cancer. Cell Death Dis. 9, 193 29416000 10.1038/s41419-017-0219-9 PMC5833354

[20] Ambros V. (2004) The functions of animal microRNAs. Nature 431, 350–355 10.1038/nature02871 15372042

[21] Shin V.Y. and Chu K.M. (2014) MiRNA as potential biomarkers and therapeutic targets for gastric cancer. World J. Gastroenterol. 20, 10432–10439 10.3748/wjg.v20.i30.10432 25132759 PMC4130850

[22] Ding L., Xu Y., Zhang W., Deng Y., Si M., Du Y. (2010) MiR-375 frequently downregulated in gastric cancer inhibits cell proliferation by targeting JAK2. Cell Res. 20, 784–793 10.1038/cr.2010.79 20548334

[23] Shang Y., Zhang Z., Liu Z., Feng B., Ren G., Li K. (2014) miR-508-5p regulates multidrug resistance of gastric cancer by targeting ABCB1 and ZNRD1. Oncogene 33, 3267–3276 10.1038/onc.2013.297 23893241

[24] Liu X., Ji Q., Zhang C., Liu X., Liu Y., Liu N. (2017) miR-30a acts as a tumor suppressor by double-targeting COX-2 and BCL9 in H. pylori gastric cancer models. Sci. Rep. 7, 7113 10.1038/s41598-017-07193-w 28769030 PMC5540978

[25] Ding L., Zhang S., Xu M., Zhang R., Sui P. and Yang Q. (2017) MicroRNA-27a contributes to the malignant behavior of gastric cancer cells by directly targeting PH domain and leucine-rich repeat protein phosphatase 2. J. Exp. Clin. Cancer Res. 36, 45 10.1186/s13046-017-0516-2 28327189 PMC5361803

[26] Jiang Z., Zhang Y., Cao R., Li L., Zhong K., Chen Q. (2017) MiR-5195-3p inhibits proliferation and invasion of human bladder cancer cells by directly targeting oncogene KLF5. Oncol. Res. 10.3727/096504016X14831120463349 PMC7841123 28109084

[27] Wang G., Cai C. and Chen L. (2016) MicroRNA-3666 regulates thyroid carcinoma cell proliferation via MET. Cell. Physiol. Biochem. 38, 1030–1039 10.1159/000443054 26937629

[28] Wang L., Lu J., Zhang H., Lyu X. and Sun Z. (2018) MicroRNA–876-;5p inhibits the progression of glioblastoma multiforme by directly targeting Forkhead box M1. Oncol. Rep. 10.3892/or.2022.8360 PMC9350973 35796017

[29] Zhang L., Wang Y., Wang L., Yin G., Li W., Xian Y. (2018) miR-23c suppresses tumor growth of human hepatocellular carcinoma by attenuating ERBB2IP. Biomed. Pharmacother. 107, 424–432 10.1016/j.biopha.2018.07.155 30103114

[30] Wu W.J., Yin H., Hu J.J. and Wei X.Z. (2018) Long noncoding RNA LINC00313 modulates papillary thyroid cancer tumorigenesis via sponging miR-4429. Neoplasma 65, 933–942 10.4149/neo_2018_180219N125 29940766

[31] Fang J., Hong H., Xue X., Zhu X., Jiang L., Qin M. (2018) A novel circular RNA, circFAT1(e2), inhibits gastric cancer progression by targeting miR-548g in the cytoplasm and interacting with YBX1 in the nucleus. Cancer Lett. 442, 222–232 10.1016/j.canlet.2018.10.040 30419346

[32] Luo X., Wang G.H., Bian Z.L., Li X.W., Zhu B.Y., Jin C.J. (2018) Long non-coding RNA CCAL/miR-149/FOXM1 axis promotes metastasis in gastric cancer. Cell Death Dis. 9, 993 30250169 10.1038/s41419-018-0969-z PMC6155366

[33] Yang Y., Qu A., Zhao R., Hua M., Zhang X., Dong Z. (2018) Genome-wide identification of a novel miRNA-based signature to predict recurrence in patients with gastric cancer. Mol. Oncol. 10.1002/1878-0261.12385 PMC6275280 30242969

[34] Zhi Y., Abudoureyimu M., Zhou H., Wang T., Feng B., Wang R. (2019) FOXM1-mediated LINC-ROR regulates the proliferation and sensitivity to sorafenib in hepatocellular carcinoma. Mol. Therapy Nucleic Acids 16, 576–588 10.1016/j.omtn.2019.04.008 31082791 PMC6514537

[35] Wang Y., Xie Y., Li X., Lin J., Zhang S., Li Z. (2018) MiR-876-5p acts as an inhibitor in hepatocellular carcinoma progression by targeting DNMT3A. Pathol. Res. Pract. 214, 1024–1030 10.1016/j.prp.2018.04.012 29724530

[36] Xu Q., Zhu Q., Zhou Z., Wang Y., Liu X., Yin G. (2018) MicroRNA-876-5p inhibits epithelial-mesenchymal transition and metastasis of hepatocellular carcinoma by targeting BCL6 corepressor like 1. Biomed. Pharmacotherapy = Biomed. Pharmacotherapie 103, 645–652 10.1016/j.biopha.2018.04.037 29679906

[37] Bao L., Lv L., Feng J., Chen Y., Wang X., Han S. (2017) MiR-876-5p suppresses epithelial-mesenchymal transition of lung cancer by directly down-regulating bone morphogenetic protein 4. J. Biosci. 42, 671–681 10.1007/s12038-017-9722-5 29229885

[38] Dong Y., Zheng Y., Wang C., Ding X., Du Y., Liu L. (2018) MiR-876-5p modulates head and neck squamous cell carcinoma metastasis and invasion by targeting vimentin. Cancer Cell Int. 18, 121 10.1186/s12935-018-0619-7 30181714 PMC6114268

[39] Xie W., Xiao J., Wang T., Zhang D. and Li Z. (2019) MicroRNA-876-5p inhibits cell proliferation, migration and invasion by targeting c-Met in osteosarcoma. J. Cell. Mol. Med. 23, 3293–3301 10.1111/jcmm.14217 30773847 PMC6484334

[40] Peng C., Huang K., Liu G., Li Y. and Yu C. (2019) MiR-876-3p regulates cisplatin resistance and stem cell-like properties of gastric cancer cells by targeting TMED3. J. Gastroenterol. Hepatol. 10.1111/jgh.14649 30843262

[41] Kurayoshi M., Oue N., Yamamoto H., Kishida M., Inoue A., Asahara T. (2006) Expression of Wnt-5a is correlated with aggressiveness of gastric cancer by stimulating cell migration and invasion. Cancer Res. 66, 10439–10448 10.1158/0008-5472.CAN-06-2359 17079465

[42] Xue Y., Zhang L., Zhu Y., Ke X., Wang Q. and Min H. (2019) Regulation of proliferation and epithelial-to-mesenchymal transition (EMT) of gastric cancer by ZEB1 via modulating Wnt5a and related mechanisms. Med. Sci. Monitor: Int. Med. J. Exp. Clin. Res. 25, 1663–1670 10.12659/MSM.912338 30829316 PMC6413562

[43] Cheng Y., Geng H., Cheng S.H., Liang P., Bai Y., Li J. (2010) KRAB zinc finger protein ZNF382 is a proapoptotic tumor suppressor that represses multiple oncogenes and is commonly silenced in multiple carcinomas. Cancer Res. 70, 6516–6526 10.1158/0008-5472.CAN-09-4566 20682794

[44] Binda E., Visioli A., Giani F., Trivieri N., Palumbo O., Restelli S. (2016) Wnt5a drives an invasive phenotype in human glioblastoma stem-like cells. Cancer Res. 28011620 10.1158/0008-5472.CAN-16-1693

[45] Kurayoshi M., Oue N., Yamamoto H., Kishida M., Inoue A., Asahara T. (2006) Expression of Wnt-5a is correlated with aggressiveness of gastric cancer by stimulating cell migration and invasion. Cancer Res. 66, 10439–10448 10.1158/0008-5472.CAN-06-2359 17079465

[46] Rauf F., Festa F., Park J.G., Magee M., Eaton S., Rinaldi C. (2018) Ibrutinib inhibition of ERBB4 reduces cell growth in a WNT5A-dependent manner. Oncogene10.1038/s41388-017-0079-x 29398709 PMC5916919

[47] Chen Z., Gao Y., Yao L., Liu Y., Huang L., Yan Z. (2018) LncFZD6 initiates Wnt/β-catenin and liver TIC self-renewal through BRG1-mediated FZD6 transcriptional activation. Oncogene10.1038/s41388-018-0203-6 PMC5992127 29535420

[48] Chiu C.S., Tsai C.H., Hsieh M.S., Tsai S.C., Jan Y.J., Lin W.Y. (2018) Exploiting Honokiol-induced ER stress CHOP activation inhibits the growth and metastasis of melanoma by suppressing the MITF and β-catenin pathways. Cancer Lett. 10.1016/j.canlet.2018.10.026 30391358

[49] Katoh M. (2018) Multi-layered prevention and treatment of chronic inflammation, organ fibrosis and cancer associated with canonical WNT/β-catenin signaling activation (Review). Int. J. Mol. Med. 42, 713–725 29786110 10.3892/ijmm.2018.3689 PMC6034925

